# Mesenchymal Stem Cell-Derived Exosomes as New Remedy for the Treatment of Neurocognitive Disorders

**DOI:** 10.3390/ijms22031433

**Published:** 2021-02-01

**Authors:** Carl Randall Harrell, Ana Volarevic, Valentin Djonov, Vladislav Volarevic

**Affiliations:** 1 Regenerative Processing Plant, LLC, 34176 US Highway 19 N Palm Harbor, Palm Harbor, FL 34684, USA; dr.harrell@regenerativeplant.org; 2 Department of Cognitive Psychology, Center for Molecular Medicine and Stem Cell Research, Faculty of Medical Sciences, University of Kragujevac, 69 Svetozar Markovic Street, 34000 Kragujevac, Serbia; ana.volarevic@medf.kg.ac.rs; 3 Institute of Anatomy, University of Bern, Baltzerstrasse 2, 3012 Bern, Switzerland; valentin.djonov@ana.unibe.ch; 4 Department of Microbiology and Immunology, Center for Molecular Medicine and Stem Cell Research, Faculty of Medical Sciences, University of Kragujevac, 69 Svetozar Markovic Street, 34000 Kragujevac, Serbia

**Keywords:** mesenchymal stem cells, exosomes, neurocognitive disorders

## Abstract

Mesenchymal stem cell (MSC)-derived exosomes (MSC-Exo) are nano-sized extracellular vesicles enriched with MSC-sourced neuroprotective and immunomodulatory microRNAs, neural growth factors, and anti-inflammatory cytokines, which attenuate neuro-inflammation, promote neo-vascularization, induce neurogenesis, and reduce apoptotic loss of neural cells. Accordingly, a large number of experimental studies demonstrated MSC-Exo-dependent improvement of cognitive impairment in experimental animals. In this review article, we summarized current knowledge about molecular and cellular mechanisms that were responsible for MSC-Exo-based restoration of cognitive function, emphasizing therapeutic potential of MSC-Exos in the treatment of neurocognitive disorders.

## 1. Introduction

Neurocognitive diseases are a heterogeneous group of disorders manifested by cognitive or behavioral dysfunction, and impairments in thinking, remembering, and reasoning, which develop as a consequence of the progressive neural loss and degeneration [1]. These diseases are age-dependent and their incident has been continuously increasing, in part because the elderly population has increased in last decades [2]. Neuroinflammation has been implicated as both an initiating and a causative factor of neurocognitive diseases [3]. As a response to microbial pathogens, innate immune cells (macrophages, dendritic cells (DCs), natural killer (NK) and natural killer T (NKT) cells), produce various pro-inflammatory cytokines and chemokines that increase permeability of the blood–brain barrier (BBB), enabling recruitment of circulating leucocytes in the central nervous system (CNS) [3]. Additionally, through the secretion of neurotrophins and immunoregulatory factors, innate immune cells provide neuroprotection, promote axonal regeneration, and maintain homeostasis in the CNS [3]. Weak immune response is incapable of eliminating infectious pathogens, while excessive immune response aggravates ongoing inflammation, resulting in the development of severe inflammation and neurodegeneration [3]. Accordingly, use of therapeutic agents, which may suppress detrimental immune response and may provide trophic support to injured neurons, is required for efficient treatment of neurocognitive diseases [3].

Mesenchymal stem cells (MSCs) are adult stem cells that reside in almost all post-natal tissues and organs, including the brain [4,5]. Several lines of evidence demonstrated that MSCs isolated from amniotic fluid (AF-MSC), dental pulp (DP-MSCs), and brain (B-MSCs) may differentiate into functional neuronal cells [4,5,6]. Additionally, MSCs, in paracrine manner, through the production of immunomodulatory factors (transforming growth factor-β (TGF-β), hepatic growth factor (HGF), nitric oxide (NO), indolamine 2,3-dioxygenase (IDO), interleukin (IL)-10, IL-6, IL-1 receptor antagonist (IL-1Ra), hemeoxygenase-1 (HO-1), prostaglandin E2 (PGE2), tumor necrosis factor alpha stimulated gene/protein 6 (TSG-6)) and pro-angiogenic factors (basic fibroblast growth factor (bFGF)), TGF-β, platelet-derived growth factor (PDGF), angiopoietin-1, placental growth factor (PGF), IL-6, monocyte chemotactic protein-1 (MCP-1), epidermal growth factor (EGF), HGF, vascular endothelial growth factor (VEGF)) regulate immune response, induce generation of new blood vessels, and provide trophic support to injured neurons, enabling enhanced repair and regeneration of neural tissue [7]. Accordingly, due to their capacity for differentiation in neural cells and due to potent immunosuppressive and angiomodulatory properties, MSCs have been explored as new therapeutic agents for the treatment of neurocognitive diseases [8].

However, several safety issues raised serious concerns about the use of MSCs in clinical settings [9]. Encapsulated structures, which contained calcifications and ossifications, were observed in the MSC-treated tissues, suggesting unwanted osteogenic and chondrogenic differentiation of transplanted MSCs under the influence of local microenvironment [9]. Additionally, combined therapy of immunosuppressive drugs and MSCs resulted in the development of respiratory and gastrointestinal infections in some patients, indicating that MSCs should not be administered together with other immunosuppressive agents [9,10]. Furthermore, several chromosomal aberrations were notice in long-term cultures of human MSC, indicating that rigorous genetic analyses of MSCs should be performed before their transplantation in patients [9,10].

Age, genetic traits, and medical history of the donor significantly affect therapeutic potential of MSCs [10]. Age-related changes, including loss of proliferation and differentiation potential and reduced therapeutic effectiveness, should be taken into account whenever MSCs are considered for autologous clinical applications in the elderly patients [10]. Similarly, in comparison with the MSCs of healthy donors, MSCs derived from patients with inflammatory or metabolic diseases showed significantly impaired proliferation and differentiation potential [10]. Although transplantation of allogeneic MSCs may circumvent age and disease-related problems, the risk of possible immunological response against allogeneic MSCs exists [11]. MSCs lack expression of major histocompatibility complex (MHC) class II and co-stimulatory molecules, but express MHC class I molecules and, therefore, may elicit strong allogeneic immune responses in MHC-class I-mismatched recipients, resulting in tissue injury and inflammation [11].

Additionally, results obtained in several clinical trials demonstrated that MSC-based immunosuppressive effects were observed in only a proportion of patients and that some MSC-treated patients, although affected by the same disease, did not respond to MSCs, while ongoing inflammation was aggravated upon injection of MSCs in some patients [9,10]. Immunophenotyping of engrafted MSCs revealed that their immunomodulatory properties depended on the cytokine milieu of the microenvironment in which MSCs were transplanted [9,11]. When MSCs are engrafted in the tissue with high levels of inflammatory cytokines, particularly interferon gamma (IFN-γ) and tumor necrosis factor alpha (TNF-α), MSCs acquire an immuno-suppressive phenotype and produce a large number of immunosuppressive factors, resulting in the inhibition of inflammatory immune cells [11]. On the contrary, when MSCs are transplanted in the microenvironment with low levels of IFN-γ and TNF-α, MSCs enhance production of pro-inflammatory cytokines and chemokines, which induce generation of inflammatory phenotype in tissue resident immune cells and attract circulating leucocytes in the tissue, contributing to the development and progression of inflammation [11].

A large number of experimental and clinical studies demonstrated that the majority of MSC-based immunoregulatory and angiomodulatory effects in the therapy of neurocognitive disorders relied on the activity of MSC-sourced bioactive factors (lipids, proteins (enzymes, cytokines, chemokines, immunoregulatory proteins, trophic, and growth factors), microRNAs (miRNAs)), which efficiently modulated immune response, induced neo-angiogenesis, and promoted repair and regeneration of injured neurons [8]. These MSC-sourced immunosuppressive and neuroprotective factors are contained within MSC-derived exosomes (MSC-Exos), which, due to their nano-sized dimension and lipid envelope, easily penetrate through the neural tissue and reach the target cells [12]. Membrane enriched in cholesterol, sphingomyelin, ceramide, and lipid raft proteins surrounds MSC-Exos’ content, protecting it from degradation. MSC-Exos express several adhesion molecules, including cluster of differentiation (CD)29, CD44, and CD73, which enable their migration to the inflamed and injured tissues. Through the direct fusion with the plasma membrane, MSC-Exos deliver their content to the cytosol of target cells, modulating their phenotype and function [5]. MSC-Exos contain nucleic acids, proteins (cytokines, chemokines), and lipids. Mass spectrometry and microarray analysis identified 4850 gene products and 4150 miRNAs within MSC-Exos. MiRNAs, particularly miR-146 and miR-21, may alter the phenotype, function, and viability of neural and immune cells and, therefore, were considered as crucially important for beneficial effects of MSC-Exos in the therapy of neuro-inflammatory diseases [5]. Since side effects related to the clinical application of MSCs were not observed in animals and patients who were treated with MSC-Exos, MSC-Exos were considered as a potential substitution for MSCs in the therapy of inflammatory and degenerative neural diseases [12].

During the past decade, a large number of experimental studies provided evidence about beneficial effects of MSC-Exos in the treatment of Alzheimer disease, Parkinson disease, post-traumatic and ischemic brain injury, schizophrenia, and autism spectrum disorders (ASD) (Table A1) [12,13,14,15,16,17]. In this review article, we emphasized their findings, indicating MSC-Exos as potentially new therapeutic agents for the treatment of neurocognitive diseases. The primary objective of this review article was to summarize current knowledge about the therapeutic potential of MSC-Exos in the treatment of neurocognitive diseases. The secondary objectives were to delineate molecular mechanisms responsible for the MSC-Exo-based alleviation of neuro-inflammation and neuro-degeneration and to identify all obstacles that limit clinical use of MSC-Exos in the treatment of cognitive and behavioral dysfunction.

## 2. Methods

An extensive literature review was carried out in December 2020 across several databases (MEDLINE, EMBASE, Google Scholar, The Cochrane Library), from 1990 to present. The systematic review was conducted according to the Preferred Reporting Items for Systematic Reviews and Meta-Analysis (PRISMA) guidelines [18]. Keywords used in the selection were: “mesenchymal stem cells”, “exosomes”, “neurocognitive dysfunction”, “degenerative diseases”, “inflammatory diseases”, “regeneration”, “immunosuppression”, “Alzheimer’s disease”, “Parkinson’s disease”, “brain injury”, “schizophrenia”, “autism spectrum disorders”. Inclusion and exclusion criteria were applied for identification of eligible studies. Articles that were not translated into the English language and did not have full text available, articles that conducted only in vitro experiments, and articles that evaluated only effects of MSC-based therapy and did not analyze the effects of MSC-sourced secretome in the treatment of neurocognitive disorders were excluded from this review article. Articles written in the English language with full text available and articles that examined MSC-Exo-dependent effects in vivo, in animals, or in patients with neurocognitive disorders were included in this review article.

C.R.H., A.V., V.D., and V.V. applied the search with the above criteria independently. A risk of bias analysis, performed by A.V. and V.V. independently, was carried out using Cochrane’s tool, Risk of Bias 2.0 (RoB 2) [19]. Each study was allocated a low, intermediate, or high risk of bias in accordance with the RoB 2.0 guidance in the five categories (selection of the reported results, measurement of the outcome, missing outcome data, deviations from intended interventions, randomization process). Various signaling questions were answered in each category and the outcomes of the signaling questions were averaged to produce an overall risk in each of the five categories [20]. An overall risk of bias was then determined for each study by the cumulative result of these five categories. A study was judged to be at high risk of bias if it was at high risk for at least one category or had some concerns for multiple categories. A study was judged to be of some concern if there were some concerns in at least one category, but not to be at high risk in any category. A study was judged to be at low risk of bias if at low risk of bias for all five categories [19,20].

**Figure 1:**
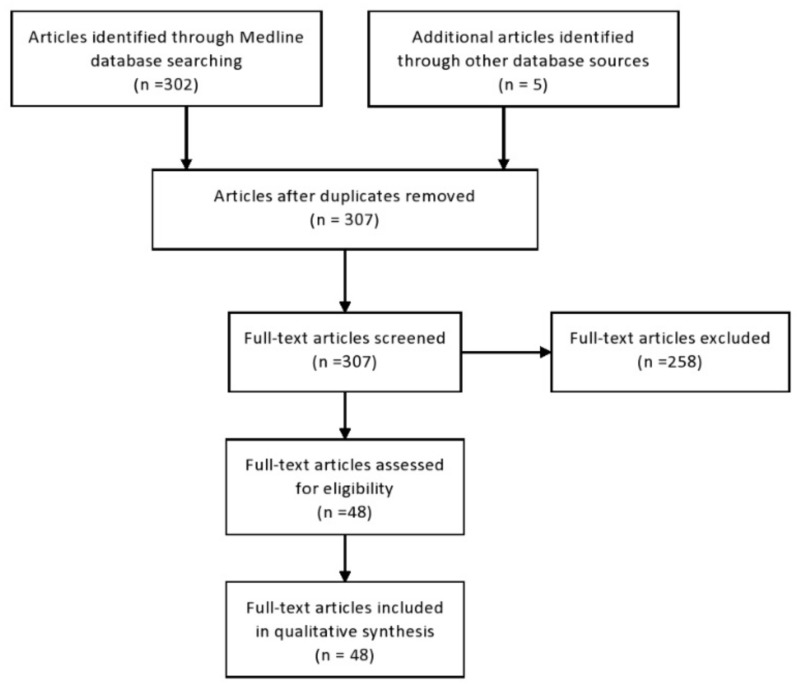
The Preferred reporting items for systematic reviews and meta-analyses (PRISMA) flow diagram. The PRISMA flow diagram shows the flow of information through the different phases of article selection.

Three hundred two articles were found from Medline. An additional five articles were retrieved from three other sources. No duplicates were identified. A total of 307 articles were retrieved, and the title and abstract of each article was screened for appropriateness. Two hundred fifty-nine articles were removed by application of the exclusion criteria. Forty-eight full-text articles were included in qualitative synthesis (Figure 1). All selected studies were assigned low risk for the domain of baseline characteristics and also had a low risk of selective outcome reporting, as well as a low risk of other sources of bias.

Study design and outcomes of each selected study were retrieved and analyzed. Details regarding the study design included the sample size, the gender and species of the animals, the animal model used, and the details of the treatment for experimental and control groups. The details of the treatment also included the tissue source of MSCs and the concentration, volume, frequency, and route of injected MSC-Exos.

All eligible studies had to delineate molecular and cellular mechanisms involved in the MSC-Exos-based therapy of neurocognitive diseases and their findings were emphasized in this review article.

## 3. Therapeutic Effects of MSC-Exos in the Treatment of Alzheimer Disease

Alzheimer disease (AD) is an incurable and progressive neurodegenerative disease manifested by memory loss, cognitive dysfunction, abnormal behavior, and impaired functions of daily living [21]. Patients with exacerbated AD usually fall into stupor state and die due to exhaustion and incapacitation [21]. The main neuropathological hallmarks of AD include accumulation of amyloidal plaques (insoluble deposits of amyloid β peptide (Aβ)) and formation of neurofibrillary tangles (intraneuronal aggregates of the hyperphosphorylated microtubule-associated protein tau), which are most usually localized in the medial temporal lobe and hippocampus of the brain [21]. Accumulated Aβ peptides inhibit transmission of the synaptic signal while intraneuronal aggregation of hyperphosphorylated tau proteins results in a disassembling of microtubules, leading to the cell death of affected neurons [21].

Although the origin of AD remains unknown, several experimental studies indicate that exosomes distribute neurotoxic molecules between neuronal cells, playing a crucially important role in AD progression [22]. MSC-Exos may use the same pathways (gap junctions, synaptic transmission, endosomal/lysosomal secretion system) to deliver MSC-sourced neuroprotective and trophic factors in injured neurons, preventing AD progression [5,12]. Accordingly, a large number of studies demonstrated beneficial effects of MSC-Exos in the treatment of AD [23,24,25,26,27,28].

Most recently, by using amyloid precursor protein/presenilin1 (APP/PS1) transgenic mice, a well-established animal model of AD, Wang and Yang demonstrated that intravenously injected bone marrow (BM)-derived MSC-Exos (BM-MSC-Exos) significantly reduced Aβ deposition and improved cognitive function recovery in APP/PS1 mice by activating sphingosine kinase (S1K)/sphingosine-1-phosphate (S1P) signaling pathway in the CNS [23]. S1K and S1P regulate metabolism of sphyngomyelin, maintain vascular integrity, and promote the development of blood vessels within the CNS, playing an important role in the delivery of neurotrophins to injured neurons [24]. Down-regulated expression of S1K and/or S1P was observed in the brain tissue samples of AD patients [25]. BM-MSC-Exos are enriched with S1P [23]. Accordingly, administration of BM-MSC-Exos reduced deposition of Aβ proteins and enhanced the expression of Neuronal Nuclei (NeuN; biomarker of neuronal cells) in the cortex and hippocampus of APP/PS1 mice [23]. Additionally, BM-MSC-Exos reduced activity of the β-site amyloid precursor protein cleaving enzyme 1 (BACE1) and down-regulated expression of presenilin-1 (PS1), which are required for the generation and subsequent accumulation of Aβ. Moreover, BM-MSC-Exos increased activity of neprilysin, Aβ-degrading enzyme that inhibits progression of AD [23]. BM-MSC-Exo-based reduction of intraneural Aβ deposition significantly improved spatial learning and memory ability of APP/PS1 mice [23]. An improvement of cognitive function was followed by increased expression of S1K and S1P and was completely diminished by intraperitoneal injection of S1K or S1P inhibitors, indicating that BM-MSC-Exo-based beneficial effects in AD relied on the activation of S1/S1P signaling pathways in neural cells [23].

Elia and colleagues also demonstrated that BM-MSC-Exo-based neuroprotection in APP/PS1 mice was based on BM-MSC-Exo-dependent activation of Aβ-degrading enzyme, neprilysin [26]. Intracerebral injection of BM-MSC-Exos significantly enhanced expression and activity of neprilysin, which reduced deposition of Aβ proteins in APP/PS1 mice. Accordingly, the total amount of dystrophic neurites was significantly lower in the cortex and hippocampus of BM-MSC-Exo-treated APP/PS1 mice compared to untreated animals [26].

Reza-Zaldivar and colleagues demonstrated that MSC-Exos restored cognitive function of AD mice in a similar manner as their parental MSCs [27]. Results obtained by Morris water maze (MWM) and Novel object recognition (NOR) test showed that injection of either MSC-Exos or MSCs efficiently alleviated cognitive impairment in AD mice [27]. Parameters that determined learning ability and memory loss showed that there was no difference in beneficial effects achieved by MSCs and MSC-Exos. MWM test showed that both MSC-Exos and MSCs significantly increased learning abilities of AD animals, while NOR test demonstrated that MSC-Exos- and MSC-treated mice exhibited similarly increased percentage of interaction time compared to the untreated AD mice, indicating that MSC-Exos were mainly responsible for MSC-based improvement of cognitive function [27].

Ma and coworkers showed that adipose tissue (AT)-derived MSC-Exos (AT-MSC-Exos) provide neuroprotection, induce neurogenesis, and ameliorate cognitive dysfunction in APP/PS1 mice [28]. After their intranasal administration, AT-MSC-Exos rapidly entered the brain and accumulated within neurons and glial cells [28]. Significantly reduced deposition of Aβ proteins and decreased activation of microglia were observed in AT-MSC-Exo-treated APP/PS1 mice [28]. Proteomics analysis showed that AT-MSC-Exos contained multiple proteins, including filamin-A, vinculin, neuropilin-1, neuroplastin, glia-derived nexin, flotillin-1, drebrin, teneurin-4, and stathmin, which induce neurogenesis and myelin formation, promote neurite outgrowth and branching, stimulate axonal growth and regeneration, and provide neuroprotection to injured neurons. RNA sequencing revealed that 1094 genes were up-regulated, while 267 genes were down-regulated in AT-MSC-Exo-treated neurons [28]. AT-MSC-Exos increased expression of PCLO, TENM1, NEXMIF genes that regulate synaptic function and improve memory function of experimental animals and decreased expression of BAD gene, which induced enhanced death of injured neurons. Accordingly, AT-MSC-Exos significantly ameliorated neurologic damage, increased total number of newly generated neurons, and efficiently rescued memory deficits in APP/PS1 mice [28].

Microglia-driven neuroinflammation aggravates the accumulation of Aβ proteins and importantly contributes to the development and progression of AD [3]. Accordingly, several studies demonstrated that MSC-Exos attenuated cognitive dysfunction of APP/PS1 mice by suppressing pro-inflammatory properties of microglia [29,30,31]. Ding and colleagues showed that umbilical cord (UC)-derived MSC-Exos (UC-MSC-Exos) modulated phenotype and function of microglia, attenuated neuroinflammation, and repaired cognitive dysfunction in APP/PS1 mice [29]. Additionally, UC-MSC-Exos significantly increased the levels of Aβ-degrading enzymes (neprylisin and insulin-degrading enzyme), which remarkably reduced Aβ deposition in the brains of experimental animals [29]. Results obtained by MWM test showed that UC-MSC-Exo-treated APP/PS1 mice had a significantly shorter mean escape latency and accomplished a larger number of platform location crossing times and a longer time spent in the target quadrant than MSC-Exo-untreated animals, suggesting that UC-MSC-Exos increased the behavioral performance by improving spatial learning and memory function of APP/PS1 mice [29]. UC-MSC-Exos attenuated the presence of Iba-1-positive microglia cells in the brain and induced polarization of microglia toward immunosuppressive M2 phenotype [29]. Significantly higher numbers of chitinase 3-like 3 (YM-1), arginase-1 (Arg-1), mannose receptors C type 1 (MRC1), and the haptoglobin/hemoglobin scavenger receptor (CD163)-expressing M2 microglia were noticed in the brains of UC-MSC-Exo-treated APP/PS1 mice than in UC-MSC-Exo-non-treated animals [29]. Accordingly, concentration of M2 microglia-derived immunosuppressive cytokines (TGF-β and IL-10) increased, while levels of M1 microglia-sourced pro-inflammatory cytokines (TNF-α and IL-1β) decreased in the peripheral blood and in the brains of UC-MSC-Exo-treated APP/PS1 mice, confirming that UC-MSC-Exos alleviated neuroinflammation and improved cognitive function of APP/PS1 mice by inducing generation of immunosuppressive phenotype in microglia [29].

In line with these findings are results obtained by Nakano and colleagues, who emphasized the important role of MSC-sourced miRNA-146 for MSC-Exo-dependent suppression of microglia and neuroinflammation in AD mice [30]. MiRNA-146 is a small, noncoding RNA molecule, which regulates inflammatory properties of microglia [30]. MSC-Exos, in miRna-146-dependent manner, inhibited TNF receptor-associated factor 6 (TRAF6) and IL-1 receptor-associated kinase 1 (IRAK1) in microglia, resulting in the down-regulated phosphorylation of nuclear factor kappa light chain enhancer of activated B cells (NF-κB) [30]. Suppression of NF-κB signaling pathway alleviated expression of inducible nitric oxide synthase (iNOS), TNF-α, IL-1β, and IL-6 genes and inhibited generation of inflammatory M1 phenotype in MSC-Exo-treated microglia [30]. Nitric oxide (NO) and pro-inflammatory cytokines (TNF-α, IL-1β), released from M1 microglia, affect synaptogenesis and impair cognitive function, resulting in the progression of AD [31]. Accordingly, by suppressing activation of M1 microglia and by promoting their differentiation in M2 immunosuppressive cells, intracerebroventricularly injected BM-MSC-Exos in miRNA-146-dependent manner alleviated neuroinflammation and improved spatial learning and memory function of APP/PS1 mice [30,31]. In addition to miRNA-146, MSC-sourced miRNA-21 was also responsible for MSC-Exos-based immunomodulation and neuroprotection in APP/PS1 mice [32]. Exosomes obtained from hypoxia-preconditioned MSCs prevented memory deficits in APP/PS1 mice by suppressing microglia activation in miRNA-21-dependent manner [32]. Significantly increased miRNA-21 corresponded to the reduced deposition of Aβ proteins, down-regulated concentration of inflammatory cytokines (TNF-α and IL-1β), reduced activation of signal transducer and activator of transcription 3 (STAT3) and NF-κB, and increased levels of anti-inflammatory cytokine IL-10 in the brains of MSC-Exo-treated APP/PS1 mice, indicating the important role of MSC-Exo-sourced miRNA-21 for beneficial effects of MSC-Exos in the treatment of AD [32].

Although MSC-Exos may cross the BBB, the majority of intravenously injected MSC-Exos accumulated in the spleen and liver [5]. Therefore, Cui and colleagues conjugated MSC-Exos with neurotropic rabies viral glycoprotein (RVG; RVG-tagged MSC-Exos) to target intravenously infused MSC-Exos to the brain of APP/PS1 mice [33]. RVG-tagged MSC-Exo exhibited improved targeting to the cortex and hippocampus of experimental animals, more efficiently prevented accumulation of Aβ proteins, and suppressed activation of microglia than RVG-nontagged MSC-Exos [33]. RVG-tagged MSC-Exo significantly reduced the levels of inflammatory cytokines (TNF-α, IL-β, and IL-6) and raised the levels of immunosuppressive IL-10 in serum samples of experimental mice. Accordingly, behavioral performance and cognitive functions of APP/PS1 mice that received RVG-tagged MSC-Exos were significantly better than learning and memory capabilities of animals that were treated with RVG-nontagged MSC-Exos [33].

Although results of preclinical studies are promising, the efficacy of MSC-Exos in restoration of cognitive function of AD patients is not demonstrated in clinical settings yet. A phase I/Ⅱ clinical trial, which will evaluate the safety and the efficacy of allogenic AT-MSC-Exos in attenuation of mild to moderate dementia, is currently recruiting AD patients for participation in the study (National Clinical Trial (NCT)04388982). Patients will be assigned to one of three experimental groups to receive 5 µg, 10 µg, or 20 µg of AT-MSC-Exos, twice a week for 12 weeks. Therapeutic effects of AT-MSC-Exos on cognitive function and quality of life of AD patients will be evaluated by Alzheimer disease assessment scale-cognitive section (ADAS-cogs) and Alzheimer disease cooperative study activities of daily living test (ADCS-ADL). This clinical trial will be conducted in China at Ruijin Hospital, affiliated to the Shanghai Jiaotong University School of Medicine. According to the estimated study completion date, the first results should be expected in April 2022.

## 4. MSC-Exos-Dependent Improvement of Cognitive Dysfunction Following Brain Injury and Ischemia

The hippocampus is essential for cognition, spatial learning, and memory [34]. Accordingly, cognitive dysfunction and memory loss develop as a consequence of hippocampal damage [34]. Traumatic injury and ischemia induce transient neurogenesis that represents a compensatory response that should promote functional recovery of the damaged neurons [35]. However, endogenous regenerative capacity of a damaged brain is very limited [35]. By providing trophic, vasoactive, and immunomodulatory factors in injured neurons and microglia, MSC-Exos inhibit detrimental immune response and promote neurogenesis and neuritogenesis [35]. Accordingly, several lines of evidence demonstrated that MSC-Exos efficiently improved cognition, learning deficiency, and memory loss in damaged and ischemic hippocampal neurons [36,37,38,39,40,41,42,43].

By using a model of acute brain injury, Niu and colleagues showed that intravenous injection of UC-MSC-Exos significantly improved cognitive function of experimental animals by regulating metabolism in hippocampal neurons [36]. Proteomic analysis revealed 67 UC-MSC-Exo-contained proteins, which promoted neurogenesis by modulating metabolism in injured neurons [36]. Among them, adiponectin was considered as the most important regulator of metabolism and neural function. Elevated levels of adiponectin, noticed in hippocampus and serum samples of UC-MSC-Exo-treated mice, corresponded to the improved cognitive function of these animals [36].

In line with these findings are results obtained by Kim and coworkers showing that intravenous injection of MSC-Exos shortly rescued pattern separation and spatial learning impairments in experimental animals with traumatic brain injury (TBI) [37]. Single intravenous injection of MSC-Exos significantly improved sensorimotor and cognitive function of rats suffering from unilateral moderate cortical contusion [38]. Remarkably, reduced hippocampal neuronal cell loss, reduced neuroinflammation, and an increased number of newly generated blood vessels and neurons were observed in the brains of MSC-Exo-treated rats with TBI [38]. MSC-Exos significantly improved cognitive function of experimental animals by suppressing activation of microglia, by preventing reactive astrogliosis, and by attenuating inflammation-induced neural degeneration. Accordingly, myelination deficits and microstructural abnormalities of the white matter were restored by MSC-Exos [39].

Zhang and colleagues also demonstrated that systemic administration of MSC-Exos improved cognitive function of rats suffering from TBI by promoting functional recovery and neurovascular remodeling [40]. Compared with the saline-treated TBI rats, MSC-Exos-treated animals showed significant improvement in spatial learning, as evidenced by MWM test [40]. Additionally, MSC-Exos attenuated inflammation and promoted neo-angiogenesis, which resulted in a significantly increased number of newly formed immature and mature neurons in dentate gyrus, indicating that MSC-Exo-induced improvement of cognitive function of TBI rats was due to the activity of MSC-Exo-sourced immunosuppressive, neurotrophic, and pro-angiogenic factors [40].

As demonstrated by Gao and colleagues [41], among various MSC-sourced molecules, miRNA-21 was mainly responsible for beneficial effects of MSC-Exos in restoration of cognitive function following brain injury. MSC-Exo-derived miRNA-21 protected neurons from apoptosis and alleviated subarachnoid hemorrhage (SAH)-induced cognitive dysfunction in experimental rats [41]. MSC-Exo-sourced miRNA-21 prevented apoptosis by inducing activation of phosphatase and tensin homolog/protein kinase B (PTEN/Akt) signaling pathway in injured neurons. MSC-Exo-dependent neuroprotection was completely abrogated by miR-21 knockdown or after the administration of PTEN/Akt inhibitor, indicating a crucially important role of miRNA-21/PTEN/Akt signaling for beneficial effects of MSC-Exos in the restoration of cognitive function after SAH [41].

Yang and colleagues showed that MSC-Exos improved postoperative cognitive dysfunction, a severe complication of cardiopulmonary bypass, by reducing hippocampal neuronal apoptosis [42]. MSC-Exo-dependent prevention of apoptosis was also responsible for improved cognitive function of rats suffering from ischemic brain injury [43]. MSC-Exos down-regulated expression of pro-apoptotic molecules (Bax, caspase-3 and -9) and enhanced expression of anti-apoptotic Bcl-2 protein, which significantly reduced the total number of dead neurons and increased neuronal density in the ischemic boundary zone [43].

Ischemic injury, oxidative stress, glucose metabolism abnormalities (hyper or hypoglycemia), changes in glutamate neurotransmission, and decreased hippocampal synaptic plasticity induce hippocampal neural injury and cognitive impairment in patients suffering from diabetes mellitus [44]. Since MSC-Exos are enriched with factors that regulate metabolism and provide trophic support to injured neurons, several studies investigated therapeutic potential of MSC-Exos for the treatment of diabetes-induced cognitive dysfunction [44,45,46]. Zhao and colleagues revealed that cognition impairment was almost completely recovered in BM-MSC-treated diabetic mice [45]. As demonstrated by Nakano and coworkers, intracerebroventricular injection of BM-MSC-Exos significantly reduced degeneration of neurons and astrocytes, as well as synaptic loss in the hippocampus of diabetic mice [44]. MSC-Exos, in miR-146a-dependent manner, down-regulated expression of IRAK1, NF-κB, and TNF-α in astrocytes and suppressed inflammation-induced damage of neurons in the hippocampus of diabetic mice, preventing the progression of diabetes-induced cognitive impairment [46].

## 5. Therapeutic Effects of MSC-Exos in the Treatment of Parkinson Disease, Schizophrenia, and ASD

Parkinson disease is a neurocognitive disorder characterized by progressive loss of dopaminergic neurons [47]. Since several experimental studies demonstrated beneficial effects of MSCs in the therapy of Parkinson disease [48], Chen and colleagues investigated whether intravenously injected MSC-Exos may induce repair and regeneration of injured dopaminergic neurons [49]. MSC-Exos easily penetrated the BBB and reached the dopaminergic neurons within substantia nigra of experimental rats [49]. MSC-Exos relieved apomorphine-induced asymmetric rotation and reduced apoptotic cell loss of dopaminergic neurons. Additionally, degenerative and necrotic changes detected in the form of deeply eosinophilic cytoplasms, accompanied by pyknosis and karyolysis, were not observed in the substantia nigra of MSC-Exo-treated rats [49]. Histological analysis revealed significant improvement in brain tissue samples of MSC-Exo-treated animals, evidenced by the presence of multipolar neurons with nucleoli and basophilic granular cytoplasms. Importantly, MSC-Exos significantly increased levels of dopamine and its metabolites (dihydroxy phenyl acetic acid and homovanillic acid) in striatum, suggesting that MSC-Exo-based therapy significantly improved function of dopaminergic neurons in animals with Parkinson disease [49].

Neurodevelopmental disorders manifested by cognitive dysfunction, increased repetitive behaviors, and deficits in communication and social interaction are defined as ASD [50]. By using Black and Tan BRachyury mice that carried the mutations at inositol 1,4,5-triphosphate receptor 3 (BTBR T+tf/J mice) which incorporate behavioral phenotype relevant to human ASD (reduced social approach, low reciprocal social interactions, and impaired juvenile play), Peters and colleagues demonstrated that intranasal administration of MSC-Exos may significantly ameliorate autism-like behavior and ASD-related symptoms [50]. Remarkably, improved male-to-male social interaction and reduced repetitive behaviors during social interaction were observed in MSC-Exo-treated BTBR mice. More complex and longer male-to-female ultrasonic vocalization was noticed in MSC-Exo-treated BTBR animals, making them more similar to healthy mice from control group. MSC-Exo-treated BTBR mice had significant improvement in the number of syllables compared to the saline-treated BTBR animals [50]. Additionally, MSC-Exos significantly improved pup retrieval behavior of female BTBR mice. While only two of 24 pups were brought back to the nest by saline-treated BTBR females, MSC-Exos-treated BTBR mothers retrieved all (18/18) pups, demonstrating significant improvement in maternal behavior [50].

Beneficial effects of intranasally injected MSC-Exos in the improvement of cognitive function were observed in phencyclidine (PCP)-treated mice, a well-established murine model of schizophrenia [51]. Tsivion-Visbord and colleagues demonstrated that intranasal delivery of MSC-Exos managed to alleviate schizophrenia-like behaviors by preserving viability of Gamma aminobutyric acid (GABA)-producing neurons and by modulating activity of neurotransmitters in the CNS [51]. Immediately after injection, the majority of MSC-Exos accumulated in the neurons of the prefrontal cortex (PFC), the brain area that is the most severely affected in schizophrenia. Significantly reduced glutamate levels in the cerebrospinal fluid and a remarkably increased number of GABA-producing neurons were observed in PFC of PCP-treated mice that received MSC-Exos. Importantly, MSC-Exos improved social interaction and disruption in prepulse inhibition in PCP-treated mice, significantly attenuating schizophrenia-like behavior [51]. Since no negative symptoms were detected following intranasal administration of MSC-Exos in mice [50,51], efficacy of this non-invasive therapeutic approach should be further explored in upcoming clinical studies for the amelioration of behavioral symptoms in patients suffering from schizophrenia and ASD.

## 6. Concluding Remarks and Future Perspectives

MSC-Exos act as an important mediator of the information exchange between MSCs and recipient cells (neurons and microglia). MSC-Exo-derived miRNAs, trophic factors, enzymes, and immunomodulatory and pro-angiogenic molecules promote neurogenesis and suppress inflammation-induced injury of hippocampal neurons, resulting in improvement of cognitive function [5,12].

Importantly, immunomodulation and neuroprotection mediated by MSC-Exos was either similar or even better than immunomodulation accomplished by their parental MSCs [5]. MSC-Exos-based effects are not affected by local tissue microenvironment. In contrast to MSCs, which alter their phenotype and function upon engraftment in different tissue microenvironments, MSC-Exos are not subject to changes in their immunomodulatory and neuroprotective properties upon diverse stimuli, indicating their potential for clinical use in the therapy of neurocognitive diseases [5,12].

Despite these promising results, several issues should be addressed before MSC-Exos can be used routinely in clinical settings. Since various number of anti-apoptotic and immunosuppressive molecules have been proposed as crucially important for the therapeutic effects of MSC-Exos in neuroprotection and restoration of cognitive function, further experimental studies should identify the exact disease-specific MSC-Exo-contained molecule(s) responsible for improved spatial learning, reduced memory loss, and functional recovery of injured hippocampal neurons. Therefore, upcoming experimental studies and clinical trials should define the exact disease-specific therapeutic dose and appropriate treatment schedule and route of MSC-Exos administration before MSC-Exos will be offered as a universal human remedy for the treatment of neurocognitive diseases.

## Data Availability

Not applicable.

## Appendix A

**Table A1:** Therapeutic effects of mesenchymal stem cell-derived exosomes in animal models of neurocognitive diseases.

Disease	Tissue Source of MSC-Exos	Therapeutic Effects	Mechanism of Action	Ref.
AD	BM	reduced Aβ deposition and improved cognitive function	activation of S1K/S1P signaling in CNS; activation of neprilysin	[23,26]
AD	AT	induced neurogenesis and ameliorated cognitive dysfunction	increased expression of PCLO, TENM1, NEXMIF genes that regulate synaptic function	[28]
AD	UC; BM	attenuated neuro-inflammation and improved cognitive function	miRNA-146 and miRNA-21-dependent polarization of microglia towards immunosuppressive M2 phenotype	[29,30,31,32]
TBI	UC; BM	improved neurogenesis and angiogenesis, attenuated neuro-inflammation	increased synthesis of adiponectin; suppression of inflammatory M1 microglia; miRNA-21-based neuroprotection; reduced hippocampal neuronal apoptosis	[36,37,38,39,40,41,42]
DM	BM	reduced degeneration of neurons and astrocytes;	miR-146a-dependent suppression of inflammatory M1 microglia	[44,45,46]
PD	BM	improved neurogenesis and cognitive function	increased regeneration of injured dopaminergic neurons	[49]
ASD	BM	improved social interaction and reduced repetitive behaviors during social interaction	suppression of inflammatory M1 microglia	[50]
SCZ	BM	improved social interaction and attenuated SCZ-like behavior	Increased viability of GABA-producing neurons	[51]

Abbreviations: Mesenchymal stem cell-derived exosomes (MSC-Exos); Alzheimer’s disease (AD); bone marrow (BM); amyloid β (Aβ); sphingosine kinase (S1K); sphingosine-1-phosphate (S1P); adipose tissue (AT); umbilical cord (UC); traumatic brain injury (TBI); diabetes mellitis (DM); Parkinson’s disease (PD); Autism spectrum disorders (ASD); Schizophrenia (SCZ); Gamma aminobutyric acid (GABA).
